# Intracoronary Infusion of Autologous CD133^+^ Cells in Myocardial Infarction and Tracing by Tc99m MIBI Scintigraphy of the Heart Areas Involved in Cell Homing

**DOI:** 10.1155/2013/582527

**Published:** 2013-07-29

**Authors:** Ubaidullo Kurbonov, Abdusamad Dustov, Alisher Barotov, Murtazokul Khidirov, Giesidin Mirojov, Zikrie Rahimov, Navjuvon Navjuvonov, Eraj Rizoev, Nasim Olimov, Alijon Goibov, Bakhtovar Karim-Zade, Mukim Rakhmatov, Suhayli Muminjonov, Azadeh Didari, Jamila Irgasheva, Oktam Bobokhojaev, Tashpulat Gulmuradov, Amu Therwath, Sohibnazar Rakhmonov, Massoud Mirshahi

**Affiliations:** ^1^Avicenna Tajik State Medical University, Dushanbe, Tajikistan; ^2^Institute of Gastroenterology, 734003 Dushanbe, Tajikistan; ^3^Tajikistan Ministry of Health, 734003 Dushanbe, Tajikistan; ^4^UMRS 872, CRC-INSERM, Université Pierre et Marie Curie, Paris-VI, Université Paris Descartes, Paris-V, 15 rue de l'Ecole de Médecine, 75006 Paris, France

## Abstract

CD133 mesenchymal cells were enriched using magnetic microbead anti-CD133 antibody from bone marrow mononuclear cells (BMMNCs). Flow cytometry and immunocytochemistry analysis using specific antibodies revealed that these cells were essentially 89 ± 4% CD133^+^ and 8 ± 5% CD34^+^. CD133^+^/CD34^+^ BMMNCs secrete important bioactive proteins such as cardiotrophin-1, angiogenic and neurogenic factors, morphogenetic proteins, and proinflammatory and remodeling factors in vitro. Single intracoronary infusions of autologous CD133^+^/CD34^+^ BMMNCs are effective and reduce infarct size in patients as analyzed by Tc99m MIBI myocardial scintigraphy. The majority of patients were treated via left coronary artery. Nine months after cell therapy, 5 out of 8 patients showed a net positive response to therapy in different regions of the heart. Uptake of Tc99 isotope and revitalization of the heart area in inferoseptal region are more pronounced (*P* = 0.016) as compared to apex and anterosptal regions after intracoronary injection of the stem cells. The cells chosen here have the properties essential for their potential use in cell therapy and their homing can be followed without major difficulty by the scintigraphy. The cell therapy proposed here is safe and should be practiced, as we found, in conjunction with scintigraphic observation of areas of heart which respond optimally to the infusion of autologous CD133^+^/CD34^+^ BMMNCs.

## 1. Introduction

Heart failure is the leading cause of death worldwide, and current therapies only delay progression of the disease. Cardiomyocytes are a stable cell population with only limited potential for renewal after injury [1, 2]. Tissue regeneration may be due to infiltration of stem cells, which differentiate into cardiomyocytes [3]. Laboratory experiments and recent clinical trials suggest that cell-based therapies can improve cardiac function [4, 5], and the implications of this for cardiac regeneration are causing great excitement. These new findings have stimulated optimism that the progression of heart failure can be prevented or even reversed with cell-based therapy [6].

Numerous studies have documented that transplantation of bone marrow derived cells following acute myocardial infarction and ischemic cardiomyopathy can lead to a reduction in infarct scar size and improvements in left ventricular function and perfusion. Furthermore, the impact of successes may be affected by quality (progenitor source) and quantity of the cells, timing [7], route (intramuscular, intracoronary) [8], and type of cardiomyopathy [4]. 

Bone marrow stem cells (BMSCs) can differentiate into multiple cell types present in the heart [9]. Following a sex-mismatched transplantation constellation heart muscle tissue analyzed after autopsy, it was revealed that mesenchymal stem cells of the BM play a pivotal role in the development of mixed chimerism of cardiomyocytes and endothelial cells following transplantation [10]. 

In several randomized studies in which BMSCs were administered by intracoronary injection, the left ventricular ejection fraction (LVEF) was measured 3–6 months following the myocardial infarction [4]; it was observed that there was a 3–12% increase (average 6%) in cardiac function [5] that has been recently in published a very informative review on ongoing clinical trials of stem cell therapy for heart diseases in USA, which stands as a good source of information.

Source of stem cell therapy for heart disease may come from progenitors from hematopoietic (BM, peripheral blood, umbilical cord blood), mesenchymal (BM, adipose tissues), skeletal (muscle), endothelial (BM, peripheral blood), and cardiac (infarct border, epicardium) cells [5]. These cells are characterized by a high potential of pluripotent activity and can participate in tissues remodeling by secretion of growth factors in an autocrine or paracrine manner. In an animal model (rat), two cell types, namely, skeletal myoblasts or CD133^+^ progenitors, led to improvement of cardiac function [11, 12]. 

Current tests such as ECG, LVEF (left ventricle ejection fraction), LVESV (left ventricle end systolic volume) and LVEDV (left ventricle end diastolic volume), are the major indicators for evaluation of efficacy of stem cell therapy. However, the efficacy of cell therapy at the *in situ* level needs to be ascertained and then alone it can be taken into account in any treatment protocol. Imaging by magnetic resonance imaging and scintigraphy allows for *in vivo* tracking of cells and can provide a better understanding and evaluation of functional impact of cardiac stem cell therapy. Among these the direct labeling of cells with isotopes and the tracking is an attractive proposal [13, 14]. 

Here, we report that CD133^+^ cells isolated from bone marrow mononuclear cells secrete a large array of regulatory proteins including several growth factors. When these cells are infused immediately in patients with coronary heart disease and postinfarction cardiosclerosis, they are able to modify revitalization of infarct scar as explored by scintigraphy. 

## 2. Material and Methods

### 2.1. Bone Marrow Specimens

 Bone marrow samples were obtained from 5 healthy individuals and 15 patients with different cardiac pathologies. All samples were obtained after informed consent of individual patients and in accordance with the rules of the revised Helsinki protocol. All participants provide their written consent to participate in this study. The ethics committees of Tajikistan Health Ministry gave their approval of the procedures fallowed and for undertaking this study.

### 2.2. Cell Preparation

 Bone marrow mononuclear cells (BMMNCs) were isolated (*n* = 5) by density-gradient centrifugation over Ficoll-400 (PAA Laboratories, Les Mureaux, France). The BMMNCs layer was collected and the monocyte/macrophage cells were eliminated by incubation of the cells with polystyrene surface. CD133^+^ was separated from BMMNCs by a magnetic bead separation method following the manufacturer's instructions (MACS; Miltenyi Biotec, France). Purity of isolated CD133^+^ was analyzed using fluorochrome-conjugated anti-CD133 monoclonal antibodies. These cell preparations contained CD34^+^ cells and their amount was quantified by immunocytochemistry using anti-CD34 monoclonal antibody (mAbs, Miltenyi Biotec, Paris, France). BMMNC-derived CD133^+^/CD34^+^ were employed in the treatment regimen described in this work. 

### 2.3. Cell Culture

 BMMNCs or isolated CD133^+^ cells were plated on 0.2% gelatin-coated wells (Sigma, Saint-Quentin Fallavier, France) and maintained in endothelial cell basal medium MV2 (ECBM MV2, Promocell, Heidelberg, Germany) supplemented with ECBM-MV2 complemented (PromoCell). At 6 days of culture, nonadherent cells were removed, new media was applied, and the culture was further maintained through days 3, 10, or 21. 

### 2.4. Cytokine Array

In order to analyze the *in vitro* secretion of bioactive proteins by bone marrow stem cells, the supernatant of BMCD133^+^ cells (*n* = 3) were analyzed using a protein cytokine array (RayBio Human Cytokine Antibody). This technique is based on the principle of  “sandwich immunoassay.” It comprises essentially of screening, in duplicate, 174 different membrane coupled anticytokines along with appropriate controls (experiments repeated 3 times). BMCD133^+^ cells (10^6^ cells per mL) were incubated in RPMI-1640 without fetal calf serum at 37°C in a humidified atmosphere of 5% CO_2_ for 24 hours. Supernatants containing cytokines were retrieved and the cytokines were allowed to couple with their specific antibodies previously immobilized on membranes. Membranes were saturated for 2 hours at room temperature with bovine serum albumin (BSA). Incubation of array membranes with supernatants (along with controls) was carried out overnight at 4°C using corresponding antibodies. After several successive washes, membranes were incubated in the presence of a mixture of antibodies and anticytokines biotinylated at 4°C overnight. Streptavidin, coupled with HRP, was added on the membranes for 2 hours at room temperature. The presence of antibody-coupled proteins was revealed by applying ECL (enhanced chemoluminescence) to the membranes, according to the recommendations of the manufacturer. Membranes were then exposed to a photosensitive film (Kodak, X-Omat AR USA). The intensity of chemiluminescence captured on the photosensitive film was measured and recorded. After subtracting the background noise, the results were expressed as a ratio of chemoluminescence intensity of experimental versus control spots. The positive control was considered as 1. Less than −2 ratio values indicated a reduction of the cytokine and a value greater than +2 indicated an increase in cytokine expression. The proteins detected by protein array from the three independent cell preparations were considered as bioactive proteins. 

### 2.5. Patients

Fifteen patients with a diagnosis of ischemic heart diseases and myocardial infarction (deferred Q-myocardial infarction without significant complications barred from 3 to 6 months) were selected. Immediately prior to implantation of the stem cells, all patients underwent coronary angiography, which was carried out on the angiographic system “Infinix CC” (Toshiba, Japan). Arteriography was performed on left coronary artery at 4–6 projections and the right coronary artery at 3-4 projections. Fluoroscopy time ranged from 2 to 9 minutes. In some of the subjects, coronary angiography revealed severe coronary artery pathology: left coronary artery trunk (4 cases) and 3 vascular lesions (7 cases). 

### 2.6. Treatment of Patients

Bone marrow was taken after standard puncture of the sternum under local anesthesia. CD133 stem cells were isolated from mononuclear cells by density gradient centrifugation using Ficoll, followed by immunomagnetic separation. Isolated cells of patients with coronary artery disease were reinjected intra-arterially into the coronary arteries under angiography with an average dose of 5 mL of suspension containing 0.8–1.5 million cells. Purified CD133^+^ positive cells were stored at 4°C in 0.9% NaCl until intramyocardial injection. Perfusion of stem cell in the coronary artery was carried out taking into account the angiographic findings and areas of myocardial ischemia: in 12 cases of the stem cells introduced directly into the left coronary artery, and in 3 cases introduced into both the coronary arteries. All the patients, in addition to their classic treatments were treated by estradiol (Estreva 0.75 mg/day for two months). 

### 2.7. Clinical Appreciations

Clinical examination and currently used tests such as ECG, EFLV (ejection fraction of the left ventricle), LVESV (left ventricle end systolic volume), and LVEDV (left ventricle end diastolic volume) were performed in order to evaluate the dynamics of myocardial perfusion in all patients with coronary heart disease and post-infarction cardiosclerosis before and after cell therapy. For 11 out of 15 of the treated patients, we carried out myocardial scintigraphy using Tc99 m with the ligand methoxyisobutylisonitrile (MIBI) after an interval of 1 and 3 months. 8 out of 11 of these patients were further investigated by scintigraphy again after 9 months.

## 3. Results

### 3.1. Isolated BMMNCs and BMMNC CD133^+^ Differentiate into Adherent Cells

BMMNCs were isolated from bone marrow of different normal donors (*n* = 5). CD133^+^ cells were isolated and their purity was found to be more than 89 ± 4% as assessed by flow cytometry. These cell preparations contained also 8 ± 5% CD34^+^ BMMNCs.

**Figure 1:**
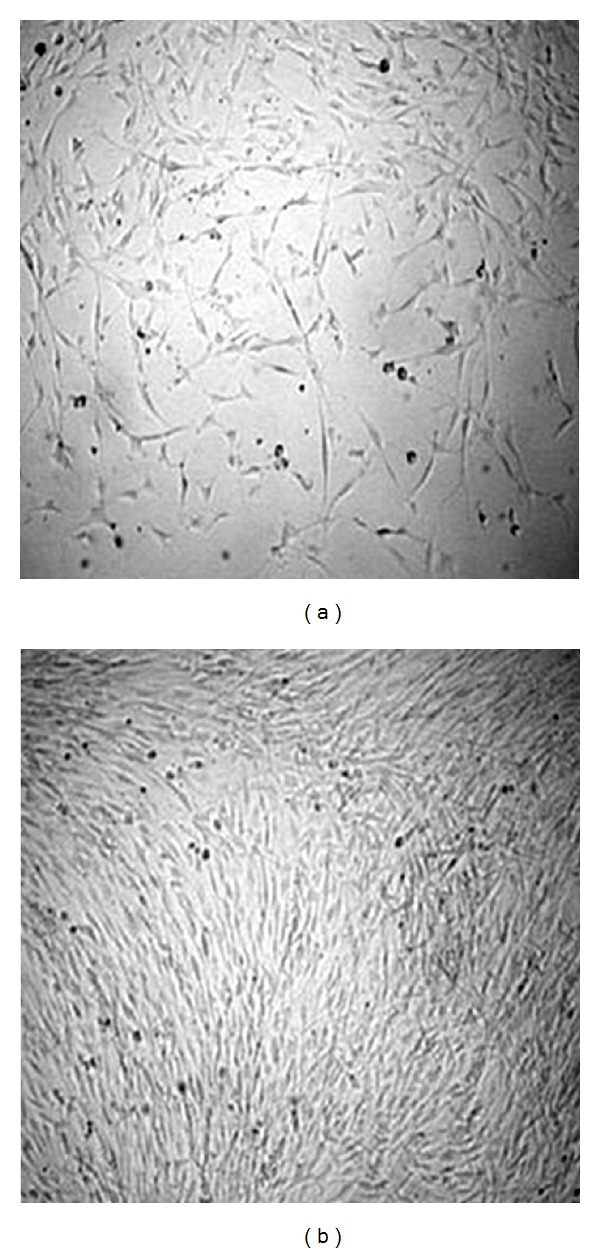
CD133^+^/CD34^+^ BMMNCs in culture after 3 days (a) and 6 days (b).

CD133^+^/CD34^+^ BMMNCs were *in vitro* cultured under specific conditions as described in Section 2.  Figure 1 presents the CD133^+^/CD34^+^ BMMNCs after 3 days (a) and 6 days (b) in culture. After 3 weeks in culture, adherent cells displaying totally different morphological aspects were observed. Indeed, certain cells were long and frayed while others were rather small (result not shown). 

### 3.2. The Adherent Cell Population Is Made Up of Various Cell Types

In order to determine the type of cells that compose the culture, immunofluorescent analysis was performed. This revealed that certain cells were positive for a specific marker of smooth muscle cells (*α*SMactin^+^), others for the specific marker of endothelial cells (CD31 and vWF^+^).

We then quantified, by counting the percentage of each cell type among the adherent cell population the ratio between stained cells/nuclei stained by DAPI (Interchem) in 5 different microscopic visual fields per patient sample, at 40X magnification. These systematic enumerations revealed that the adherent cell population was composed of 23 ± 5% of anti-*α*-smooth muscle cells and 25 ± 5% of endothelial cells. Interestingly, the remaining adherents' cell population 52 ± 7% was systematically negative for the 2 markers tested.

### 3.3. The Adherents Cells Secrete Bioactive Proteins

After 3 days of culture, three samples of CD133^+^/CD34^+^ BMMNCs cells from 3 different donors were incubated with conditioned medium at 37°C for 36 h. The supernatants were tested by Ray-Bio protein array. As presented in Table 1, CD133^+^/CD34^+^ BMMNCs secrete *in vitro* important bioactive proteins such as cardiotropin-1, angiogenic factors (angiogenin, angiopoitein-2, basic fibroblast growth factor, placenta growth factor, (VEGF) vascular endothelial growth factor-121, VEGF-165, and VEGF-D), neurogenic factors (agouti-related protein, brain-derived neurotropic factor, human ciliary neurotrophic factor, basic nerve growth factor, amphiregulin, neurotrophin-3, and 4, activin A and prolactin), morphogenetic proteins (bone morphogenetic protein, BMP-4, 5, 6, and 7), and several proinflammatory and remodeling factors. Several cytokines were absent however among the bioactive proteins tested (results not shown). 

### 3.4. Use of CD133^+^/CD34^+^ BMMNCs for Intracoronary Infusions

For cell therapy, isolated CD133^+^/CD34^+^ BMMNCs were injected into the coronary arteries via the femoral artery. Patients were examined after 3, 6, and 9 months. 

The results of clinical examinations of patients at these two time intervals showed a net improvement beginning three months and also six and nine months after infusion. Examination indicated that the general physical state of patients improved, such as effort tolerance, physical endurance, and overall autonomy. In addition, the treated patients had a better psychic state “the effect of a perfect action” as compared to the control. One of the patients in the treated group died as also two others in the control group (one of them by accident).

### 3.5. Infarct Scar Size Was Reduced after Intra coronary Stem Cell Infusion

**Figure 2:**
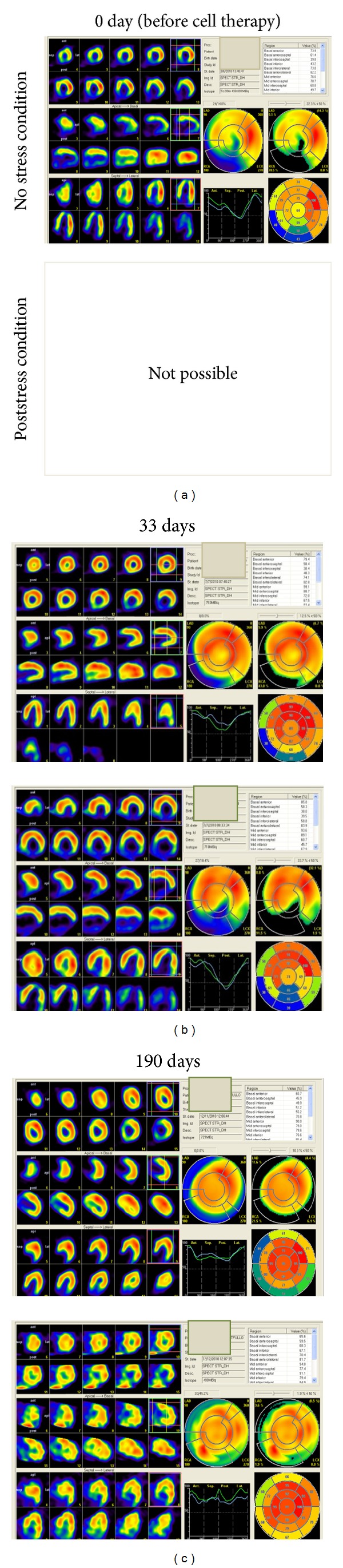
Kinetic of the areas that benefit from myocardial revitalization after cell therapy carried out by myocardial scintigraphy before and after 33 and 190 days. The strong uptake of Tc99 isotope in the inferoseptal region of the heart in poststress condition showed by arrow. (black, no perfusion; blue-green-yellow-red, increasing perfusion).

**Figure 3:**
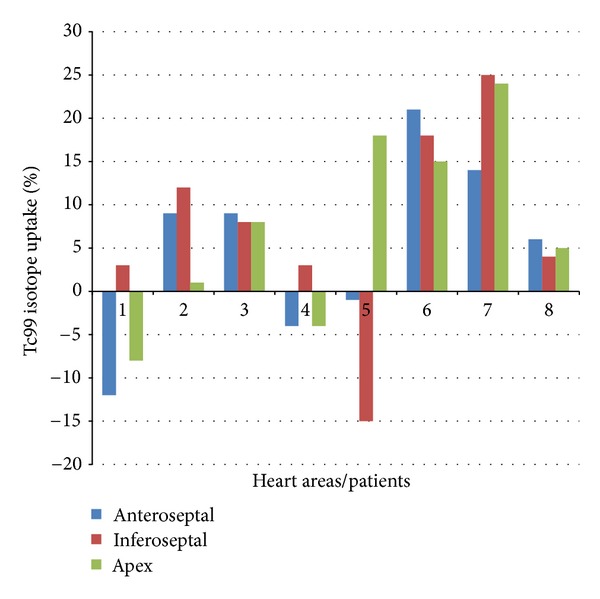
Tc99 isotope uptake in different areas of the heart I poststress condition, quantitative measurements of activity in the area at risk (anteroseptal, inferoseptal, and apex) expressed as the difference between postoperative perfusion and preoperative perfusion.

Myocardial Tc99m MIBI scintigraphy in 11 patients was performed to evaluate the dynamics of myocardial perfusion.  Figure 2 presents the kinetics of uptake of Tc99 isotope in cardiac areas before and after stress (after 33 and 190 days of cell therapy). Scintigraphy investigations of heart in Eight out of 11 (Table 2) patients after 1 and 3 months of cell perfusion in left coronary artery (*n* = 7) and right coronary artery (*n* = 1) showed considerable increase in stable perfusion, as monitored by indicators. The continuing progress was again noted after a period of 9 months. In these patients, high inclusion of Tc99 (%) in the region of anteroseptal (Table 3(a)), inferoseptal (Table 3(b)), and apex (Table 3(c)) before cell therapy (rest and stress) and after cell therapy (stress) as determined. Images were assessed by quantitative measurements of activity in the area at risk and expressed as the difference between postoperative perfusion and preoperative perfusion. After a single infusion of stem cells (Figure 3), we noted a difference in Tc99 isotope uptake of myocardium in 5 out of 8 patients for Anteroseptal, 7 out of 8 patients for Inferoseptal and 6 out of 8 patients for apex regions. These results show that revitalization of the myocardium is not the same in different anatomic parts of the heart. 

As presented in Table 4, the treated patients were divided into two categories: (A) responders and (B) non responders. High inclusion of Tc99, after cell therapy, changed significantly only in inferoseptal region (*P* = 0.016) in responder patients (*n* = 5). In non responders, inclusion of Tc99 in different regions of the heart was not significantly different between preoperative and postoperative perfusion under stress conditions (*n* = 3). These results suggest that the inferoseptal zone is a good target for stem cell therapy. In 7 patients out of 8, uptakes of isotope in all parts of the heart were increased. 

**Figure 4:**
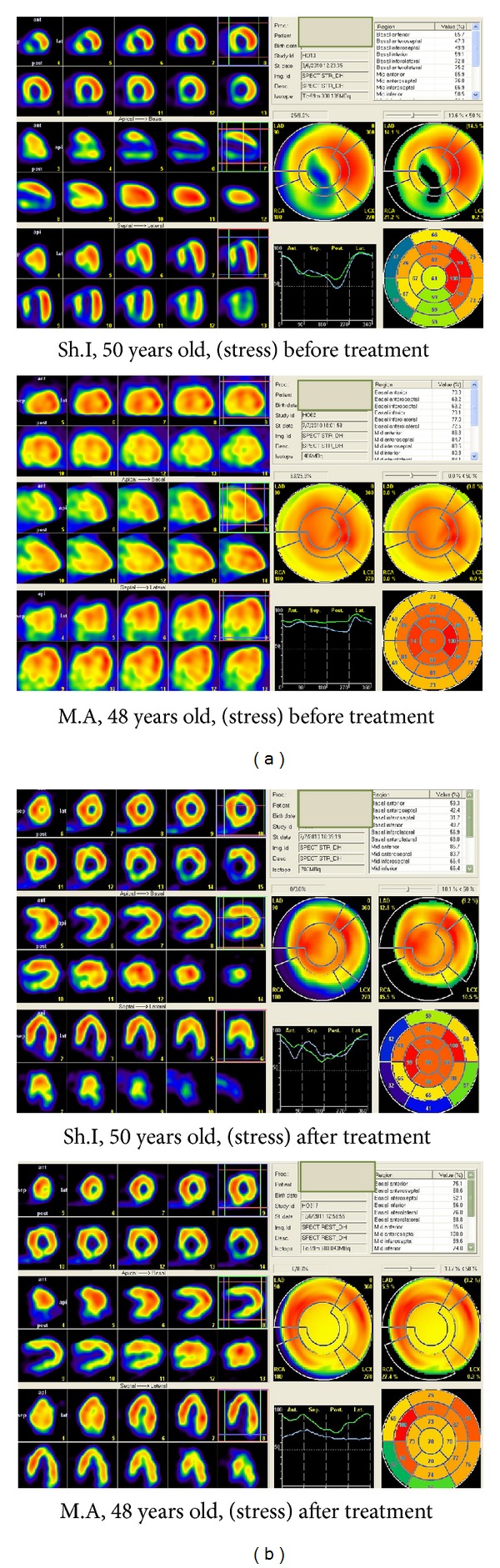
The areas that benefit from myocardial revascularization after single cell therapy in two patients, carried out by myocardial scintigraphy (post stress condition) before treatments and after 9 months (black, no perfusion; blue-green-yellow-red, increasing perfusion).

The areas that benefit from a myocardial revitalization, 9 months after cell therapy in case of two patients are presented in Figure 4. One of these two (Sh.I., patient no. 2), presented a minimal uptake of Tc99 before treatment in anterior, septal, anteroseptal and inferoseptal regions (Figure 4(a)). After treatment, the regions of anterior and septal of the heart were strongly revitalized (Figure 4(b)). This patient was treated by 52 × 10^4^ CD133^+^/CD34^+^ BMMNCs, and the cells were introduced by left coronary artery (LCA). In the second patient (M.A, patient no. 7), the septal, anteroseptal, and inferoseptal regions were damaged (Figure 4(a)). After cell therapy, revitalization was observed in all these regions (see Figure 4(b)). This patient was treated by 68 × 10^4^ CD133^+^/CD34^+^ BMMNCs, and the cells were introduced by right coronary artery (RCA). These results clearly demonstrate a positive effect of cell therapy as ascertained by scintigraphic diagnostics with Tc99 m in patients with CHD and post-infarction cardiosclerosis during 9 months monitoring periods following cell therapy. These patients continue to make progress as of present.

## 4. Discussion

Bone marrow CD133-positive (CD133^+^) cells possess strong hematopoietic and angiogenic capacity and can differentiate into several tissue types such as adipocyte, chondrocyte, osteocyte, neurocyte, and myocyte. We tested the feasibility, safety, and functional effects of the use of enriched CD133^+^ progenitor cells after intracoronary administration in patients with coronary heart disease and post-infarction cardiosclerosis. 

We noted that when we use magnetic microbead technique for enrichment of CD133^+^ cells, the cell preparation also contained CD34^+^ progenitor cells. CD133^+^/CD34^+^ BMMNCs with specific medium can differentiate into several cells types such as endothelial cells, adipocyte, osteocyte, neurocyte, and myocyte [15] (results not shown). In this study, only differentiation of CD133^+^/CD34^+^ BMMNCs to endothelial and myofibroblasts with *α*SMactin^+^ cells was demonstrated. These results confirm the earlier observation concerning the pluripotent nature of CD133^+^/CD34^+^ BMMNCs. 

We analyzed the secretion of bioactive proteins of CD133^+^/CD34^+^ BMMNCs *in vitro*. The proteins secreted are angiogenic and neurogenic factors, morphogenetic proteins and several growth factors. One of the interesting bioactive proteins secreted by these cells is cardiotrophin-1 (CT-1) which is a member of the interleukin-6 type cytokine family. These cytokines mediate overlapping pleiotropic actions in a variety of cell types including cardiac myocytes, hepatocytes, megakaryocytes, osteoclasts, and neuronal cells. It is important to note that CT-1 was shown to specifically protect the cardiac myocytes from ischemic damage [16]. The role of CD133 in undertaking repair of heart regions is multistep and the intervention of several factors during the process undeniably reinforces our choice of CD133 for the treatment protocol in heart disease. 

CD133^+^/CD34^+^ BMMNCs have also a strong pro-angiogenic capacity (Table 1(a)) as they secrete several factor such as angiogenin, angiopoitein-2, VEGF-121 (vascular endothelial growth factor) and 165, VEGF-D, PLGF (placenta growth factor) and b-FGF (basic fibroblast growth factor). These factors could be involved in angiogenesis/revascularization after cell therapy [17]. 

We have shown that CD133^+^/CD34^+^ BMMNCs also produce several pro-inflammatory factors including chemokines I-309 (CCL-1), MCP-1 (CCL-2), MIP-1 family (CCL-4), RANTES (CCL-5), interleukin-1 receptor antagonist (IL-1ra), CXCL-16, MIF, and sTNFR-1. These proinflammatory factors secreted by the stem cells (Table 1(b)) in all probability intervene in tissue remodeling in the infarct zone. 

Bone marrow stem cells secrete several interesting matrix metalloproteinase (MMPs) such as MMP1 (collagenase), MMP3 (stromelysin), MMP9 (gelatinase), MMP13 (collagenase), and their inhibitors (TIMPs): TIMP1, TIMP2, and TIMP4. MMPs belong to a larger family of proteases known for their role in remodeling extracellular matrix and affecting cell behaviors such as cell proliferation, migration, differentiation and angiogenesis. The tissue inhibitors of metalloproteinases are naturally occurring proteins that specifically inhibit matrix metalloproteinases and contribute towards maintaining a balance between matrix destruction and matrix formation (Table 1(c)). The presence of these specialized family proteins, secreted by stem cells, goes in favor of their importance in matrix guided remodeling of tissue substrates. 

As presented in Tables 1(d) and 1(e), CD133^+^/CD34^+^ BMMNCs secrete neurophilic and bone morphogenetic bioactive proteins. The neurophilic factors essentially AGRP (agouti-related protein), BNDF (brain-derived neurotrophic factor), CNTF (human ciliary neurotrophic factor), AREG (schwannoma-derived growth factor, Amphiregulin) is a growth factor as well as a mitogen for astrocytes, Schwann cells, and fibroblasts [18], b-NGF (basic nerve growth factor), NT-3 (neurotrophin-3 ), NT-4 (neurotrophin-3 ), activin A, and prolactin (Table 1(d)). These factors promote neurogenesis and neural cell differentiation [19]. These factors are of importance in establishing/reestablishing axis of control between the myocardium and the nerve innervations. 

The morphogenetic proteins produced by CD133^+^/CD34^+^ BMMNCs are BMP-4, BMP-5, BMP-6, and BMP-7 (Table 1(e)). These proteins constitute a group of important morphogenetic signals, needed in orchestering tissue architecture throughout the body [20]. They are the major actors during embryonic development, particularly in embryonic patterning and early skeletal formation [21]. They also participate in vasculature-guided neuronal migration under both normal and pathological conditions [22]. Once again, the coordinated action of angiogenic, neurogenics and morphogenic provided by CD133^+^/CD34^+^ BMMNCs seems necessary in the cellular mechanism leading to recovery of damage in the heart. 

For regeneration of myocardium in heart disease, several strategies with different progenitor cells were proposed (see Section 1). Among these, use of CD133^+^/CD34^+^ BMMNCs seemed an attractive candidate. The fact is that the stem cells provide the molecular and cellular properties towards altering the microenvironment with which they come in contact seems unquestionable. They seem to have an inherent capacity for reprogramming of microenvironment at cellular and molecular levels. The cell-cell-matrix interaction is a necessary phase leading to tissues repair of injury/scar. It is thus obvious that CD133^+^/CD34^+^ BMMNCs stem cell therapy in heart disease is the one of choice that can be proposed to patients with minimal risk. 

In this work, we therefore used CD133^+^/CD34^+^ BMMNCs in cardiac tissue remodeling in patients with coronary heart diseases and postinfarction cardiosclerosis. In 12 patient stem cells were introduced directly into the left coronary artery and in 3 cases: into both the left and the right coronary arteries. However in practice there were fewer injections of stem cells into the right coronary artery on account of frequent occurrence of occlusion of this blood vessel. In such cases, the introduction of the total dose of stem cells via the left coronary artery contributed to the passage of these cells in the ischemic zone of the right coronary artery through the collateral interconnections.

 In 7 out of 8 patients, the cells were injected in left anterior descending coronary artery and only in one patient the cells were infused in right coronary artery. The patients were monitored during 1 to 9 months. We attempted using scintigraphy to follow up the effect of introduction of stem cells in the different areas of the heart. This study therefore confines to an *in situ* observation of heart condition after cell infusion. 

Intracoronary stem cell infusion yielded a reduction of Infarct scar size. This revitalization that was observed in 11 of 15 patients (results not shown) may be due to an angiogenesis process after CD133^+^/CD34^+^ BMMNCs therapy or cardiomyogenesis, or both. CD133^+^ cells can differentiate into both myocytes and endothelial cells, but CD34^+^ cells may be differentiate to give only endothelial cells via angioblast maturation. 

Nine months after cell therapy, 5 out of 8 patients showed a net positive response to therapy in different regions of the heart evaluated by scintigraphy. Uptake of Tc99 isotope and revitalization of the heart area in inferoseptal region are more pronounced as compared to the apex and anterospetal regions after intracoronary injection of the stem cells. In addition, we noted that uptakes of TC99 isotope in 2 patients before and after cell therapy 9 months after treatment. They showed that cell therapy by 2 different routes (LCA or RCA) was able to revitalize different areas of the heart and suggesting that stem cell therapy can generate the collateral vasculature for irrigation of heart areas. After 9 months, a better psychic state was also noted in all treated patients. 

In conclusion, in this nonrandomized study, we indicate that CD133 BMMNCs secrete important bioactive proteins and can be an excellent choice for cell therapy. Intracoronary infusion of autologous CD133^+^/CD34^+^ BMMNCs reduces infarct size in patient with coronary heart disease and post infarction cardiosclerosis. The cell therapy approach proposed here should be practiced in conjunction with scintigraphy observation of areas of heart which respond optimally to the infusion of autologous CD133^+^/CD34^+^ BMMNCs.

## Figures and Tables

**Table 1:** Biological classification of the bioactive proteins secreted by the primo culture of CD133^+^/CD34^+^ BMMNCs (87 ± 4%/8 ± 5%) after 36 h in conditioned culture medium.

Growth factors secreted by CD133^+^/CD34^+^ BMMNCs	
(a) Proangiogenic factors	
Angiogenin	
Angiopoitien-2	
b-FGF (basic fibroblast growth factor)	
PLGF (placenta growth factor)	
VEGF-121 (vascular endothelial growth factor-121)	
VEGF165 (vascular endothelial growth factor-165)	
VEGF-D (vascular endothelial growth factor-D)	

(b) Proinflammatory factors	
I-309 (CCL-1 (C-C motif) ligand-1)	
MCP-1 (CCL-2 (C-C motif) ligand-2)	
MIP-1a (CCL-4 (C-C motif) ligand-4)	
RANTES (CCL-5 (C-C motif) ligand-5)	
IL-1ra (interleukin-1 receptor antagonist)	
CXCL-16 (C-X-C motif) ligand-16	
MIF (macrophage migration inhibitory factor)	
sTNFR-1 (soluble tumor necrosis factor receptor-1)	

(c) MMPs-TIMPs	
MMP-1 (matrix metalloproteinase-1)	
MMP-3 (matrix metalloproteinase-3)	
MMP-9 (matrix metalloproteinase-9)	
MMP-13 (matrix metalloproteinase-13)	
TIMP-1 (tissue inhibitor of metalloproteinases-1)	
TIMP-2 (tissue inhibitor of metalloproteinases-2)	
TIMP-4 (tissue inhibitor of metalloproteinases-4)	

(d) Neurophilic factors	
AGRP (agouti-related protein)	
BDNF (brain-derived neurotrophic factor)	
CTNF (human ciliary neurotrophic factor)	
b-NGF (basic nerve growth factor)	
AREG (amphiregulin)	
NT-3 (neurotrophin-3)	
NT-4 (neurotrophin-4)	
Activin A (promotes neural cell differentiation)	
Prolactin (promotes neurogenesis in maternal and foetal brains)	

(e) Morphogenetic proteins	
BMP-4 (bone morphogenetic protein-4)	
BMP-5 (bone morphogenetic protein-5)	
BMP-6 (bone morphogenetic protein-6)	
BMP-7 (bone morphogenetic protein-7)	

**Table 2:** characteristics of patients.

Samples	Patients	B.D	Diseases	Routes	I.C. infused cells × 10^4^
1	S.Sh.	1961	MI	LCA	60
2	Sh.I.	1944	MI	LCA	52
3	F.K.	1951	MI	LCA	116
4	A.L.	1950	MI	LCA	72
5	K.A.	1947	MI	LCA	80
6	B.M.	1948	MI	LCA	116
7	M.A.	1953	MI	RCA	76
8	M.Sh.	1966	MI	LCA	68

B.D: birth day, MI: myocardial infarction, LCA: left coronary artery, RCA: right coronary artery, intracoronary (I.C.) infused cells.

**Table:** (a) Anteroseptal regions of heart

No. of months	Patients	Anteroseptal before		Anteroseptal after treatment 9 months stress %	Evaluation stress %
		Rest %	Stress %		
1	S.Sh	62,0	81,0	69,0	−12
2	Sh.I	47,0	73,0	82,0	9
3	F.K	58,0	57	66,0	9
4	A.L	46,0	60,0	56,0	−4
5	K.A	55,0	73,0	72,0	−1
6	B.M	61,0	63,0	84,0	21
7	M.A	33,0	43,0	57,0	14
8	M.Sh	41,0	47,0	53,0	6

**Table:** (b) Inferoseptal regions of heart

No. of months	Patients	Inferoseptal before		Inferoseptal after treatment 9 months stress %	Evaluation stress %
		Rest %	Stress %		
1	S.Sh	50,0	63,0	66,0	3
2	Sh.I	51,0	50,0	62,0	12
3	F.K	39,0	58	66,0	8
4	A.L	35,0	35,0	38,0	3
5	K.A	61,0	66,0	51,0	−15
6	B.M	71,0	57,0	75,0	18
7	M.A	45,0	43,0	68,0	25
8	M.Sh	41,0	50,0	54,0	4

**Table:** (c) Apex regions of heart

No. of months	Patients	Apex before		Apex after treatment 9 months stress %	Evaluation stress %
		Rest %	Stress %		
1	S.Sh	51,0	67,0	59,0	−8
2	Sh.I	61,0	56,0	57,0	1
3	F.K	52,0	41,0	49,0	8
4	A.L	43,0	45,0	41,0	−4
5	K.A	58,0	48,0	66,0	18
6	B.M	70,0	59,0	74,0	15
7	M.A	55,0	44,0	68,0	24
8	M.Sh	56,0	59,0	64,0	5

**Table 4:** Scintigraphic investigations of heart in (A) in responders (5 out of 8 patients) and (B) in nonresponders (3 out of 8 patients) before treatment and after a period of 9 months. Inclusion of Tc99 in the regions of anteroseptal, inferoseptal, and apex before and after cell therapy (stress condition) increased significantly only in inferoseptal region (*P* = 0.016) in responder patients. In nonresponders, inclusion of Tc99 in different regions of the heart was not significantly different between preoperative and postoperative perfusions under stress conditions.

(A)	Before treatment	After treatment	*P* value

Anteroseptal	56,60 ± 12,12	68,40 ± 14,55	0,194
Inferoseptal	51,60 ± 6,11	65,00 ± 7,75	0,016
Apex	51,80 ± 8,64	62,10 ± 9,71	0,106

(B)	Before treatment	After treatment	*P* value

Anteroseptal	71,33 ± 10,60	65,00 ± 6,7	0,510
Inferoseptal	50,33 ± 15,50	50,33 ± 12,0	1,00
Apex	49,67 ± 5,69	54,60 ± 12,6	0,566
